# Astragaloside IV Alleviates Ammonia-Induced Apoptosis and Oxidative Stress in Bovine Mammary Epithelial Cells

**DOI:** 10.3390/ijms20030600

**Published:** 2019-01-30

**Authors:** Fengge Wang, Yun Zhao, Shuxiong Chen, Lu Chen, Liting Sun, Maosheng Cao, Chunjin Li, Xu Zhou

**Affiliations:** College of Animal Sciences, Jilin University, Changchun 130062, China; wangfg16@mails.jlu.edu.cn (F.W.); zhaoyun13@mails.jlu.edu.cn (Y.Z.); chensx14@mails.jlu.edu.cn (S.C.); luchen@jlu.edu.cn (L.C.); sunlt16@mails.jlu.edu.cn (L.S.); caoms18@mails.jlu.edu.cn (M.C.)

**Keywords:** astragaloside IV, ammonia, apoptosis, oxidative stress

## Abstract

Ammonia is one of the major toxic components of metabolites in blood and tissues of high-producing dairy cows and could affect the health of bovine mammary glands. Bovine mammary epithelial cells are sensitive to oxidative stress induced by intensive cell metabolism. In our previous study, we found that ammonia could induce oxidative stress, apoptosis and inflammatory responses in bovine mammary epithelial cells. In the present study, the cytoprotective effects of astragaloside IV against ammonia in vitro were explored. The results demonstrated that pretreatment of MAC-T cells with astragaloside IV could potently suppress the increase in the level of intracellular reactive oxygen species (ROS) and the rate of cell apoptosis, inhibit the ammonia-induced inflammatory responses, and rescue the decrease of cell viability. Astragaloside IV prevented ammonia-induced endoplasmic reticulum stress. Astragaloside IV also significantly suppressed the levels of BAX, caspase 3 and p53 phosphorylation in ammonia-induced MAC-T cells. Nuclear factor erythroid 2-related factor 2(Nrf2) was essential for cytoprotective effects of astragaloside IV in MAC-T cells, as knockdown of Nrf2 dramatically abolished the prosurvival effects of astragaloside IV on treated cells. Furthermore, the PI3K/AKT and ERK/MAPK pathways were responsible for the induction of Nrf2 by astragaloside IV. In conclusion, astragaloside IV played a beneficial role against ammonia-induced damage of MAC-T cells. This provides a cue for future study to use astragaloside IV as a protective and curative agent against ammonia exposure of mammary glands in dairy cows.

## 1. Introduction

Ammonia, produced mainly from the deamination of amino acids and glutamine, is one of the major toxic components in blood and tissues that may affect bovine health [1]. In addition, it has been shown that cows fed with high protein feed to maximize milk yield exhibit elevated ammonia concentrations in their tissues and blood [2]. A number of papers involving both in vivo and in vitro studies have reported that elevated concentrations of ammonia in animals or culture medium can induce cellular apoptosis and oxidative stress [1,3]. In a previous study, we found that ammonia could induce oxidative stress and apoptosis in bovine mammary epithelial cells [3]. During oxidative stress, high levels of ROS exert damage on the physiological functions of proteins, lipids, nucleic acids and other macromolecular substances in cells. It has been shown that oxidative stress might be associated with some dairy cow diseases, such as udder edema, retained placenta, mastitis and suboptimal reproductive performance [4]. 

Astragaloside IV (AS IV), one of the major and active components of *Astragalus membranaceus* (Fisch) Bunge, has been shown to have a strong anti-oxidative effect by removing free radicals, decreasing lipid peroxidation [5]. Astragaloside IV mops up radicals by activating antioxidant pathways. Diverse pharmacological effects of astragaloside IV have been found such as anti-inflammation [6], anti-diabetes [7], anti-hypertension [8], and myocardial protection, anti-heart failure [9], and anti-infarction effects [10]. Anti-oxidative effects of astragaloside IV have been reported in both in vitro and in vivo studies [11,12]. However, its anti-oxidative role in bovine mammary epithelial cells induced by ammonia has not been well understood. In the present study, using an in vitro model, we investigated the protective role and mechanisms of astragaloside IV against ammonia-induced oxidative stress and apoptosis of bovine mammary epithelial cells. 

## 2. Results

### 2.1. Effect of Astragaloside IV on Ammonia-Induced Bovine Mammary Epithelial Cell Death

**Figure 1:**
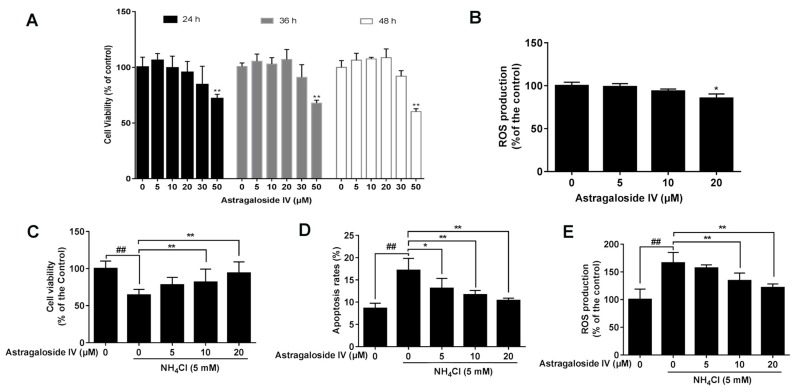
The protective effects of astragaloside IV against ammonia -induced cell death and ROS production in MAC-T cells. (**A**) The effects of different concentrations of astragaloside IV (0, 5, 10, 20, and 50 μM) for 24 h, 36 h or 48 h on the viability of MAC-T cells. The cell viability was measured by CCK-8 assay. The data are shown as mean ± SD. *n* = 6. **, *p* < 0.01. (**B**) The effects of different concentrations of astragaloside IV (0, 5, 10, and 20 μM) for 24 h on the ROS concentration of MAC-T cells. The data are shown as mean ± SD. *n* = 4. *, *p* < 0.05. The MAC-T cells were pretreated with different concentrations of astragaloside IV (0, 5, 10 and 20 μM) for 4 h, followed by NH_4_Cl (5 mM) treatment for 24 h. The cell viability (**C**), the percentage of cell apoptosis (**D**) and ROS concentration (**E**) were measured. The data are shown as mean ± SD. *, *p* < 0.05; **, *p* < 0.01. ## indicates a significant difference from untreated cells (*p* < 0.01).

Cells treated with astragaloside IV at various concentrations (0, 5, 10, and 20 μM) for different times showed no effect on bovine mammary epithelial cell growth (Figure 1A). However, astragaloside IV at a concentration of 50 μM significantly decreased the cell viability. We predicted that a high concentration of astragaloside IV might have a toxic effect. In addition, astragaloside IV at a concentration of 20 μM significantly decreased the concentration of ROS (Figure 1B). Pretreatment of cells with astragaloside IV at concentrations of 10 and 20 μM before exposure to ammonia significantly increased cell viability (Figure 1C) and decreased the percentage of apoptotic cells (at the concentrations of 5, 10, and 20 μM) (Figure 1D) and ROS level (at the concentrations of 10, and 20 μM) (Figure 1E) compared to the treatment with ammonia alone. The results showed that astragaloside IV alleviated ammonia-induced cell death.

### 2.2. Effects of Astragaloside IV on mRNA Expressions of Apoptosis-Related Genes Induced by Ammonia in Bovine Mammary Epithelial Cells

**Figure 2:**
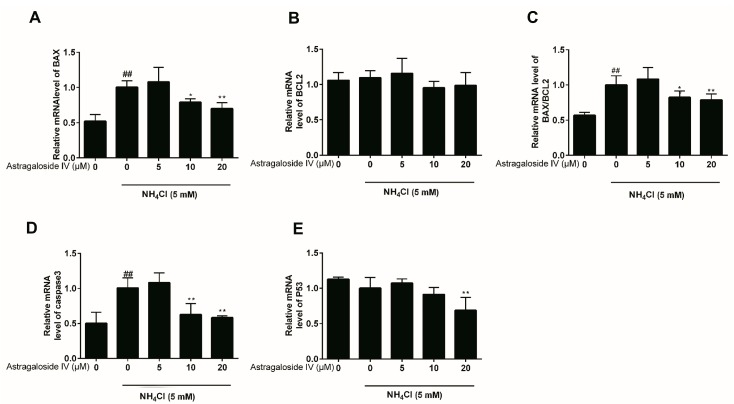
The protective effects of astragaloside IV on mRNA expressions of genes (*BAX*, *caspase 3*, *BCL2*, *BAX/BCL2* and *p53*) induced by ammonia in MAC-T cells. The MAC-T cells were pretreated with different concentrations of astragaloside IV (0, 5, 10 and 20 μM) for 4 h, followed by NH_4_Cl (5 mM) treatment for 24 h. Apoptosis-related genes (*BAX* (**A**), *BCL-2* (**B**), *BAX/BCL2* (**C**), *caspase3* (**D**), and *p53* (**E**)) were analyzed by quantitative real-time PCR. The data are shown as mean ± SD. *n* = 4. *, *p* < 0.05; **, *p* < 0.01. ## indicates a significant difference from untreated cells at *p* < 0.01.

To further analyze the mechanisms of astragaloside IV inhibiting ammonia-induced apoptosis in the MAC-T cells, genes involved in cell apoptosis were detected using RT-PCR. Consistent with our previous research [3], ammonia significantly increased the expressions of mRNAs of BAX, caspase 3 and the ratio of BAX/BCL2 in MAC-T cells. However, the expressions of BAX, BAX/BCL2 and caspase 3 induced by ammonia were suppressed significantly by the pretreatment of the cells with astragaloside IV (10 and 20 μM) (Figure 2). In contrast, there were no significant differences in the expression of mRNAs of BCL2 and p53 when the concentrations of astragaloside IV were 5 μM and 10 μM. However, when the concentration of astragaloside IV was 20 μM, the mRNA expression of p53 was significantly decreased compared to both the control group and the cells treated with ammonia alone.

### 2.3. Effects of Ammonia and Astragaloside IV Treatment on p53 Signaling Pathways in Bovine Mammary Epithelial Cells

**Figure 3:**
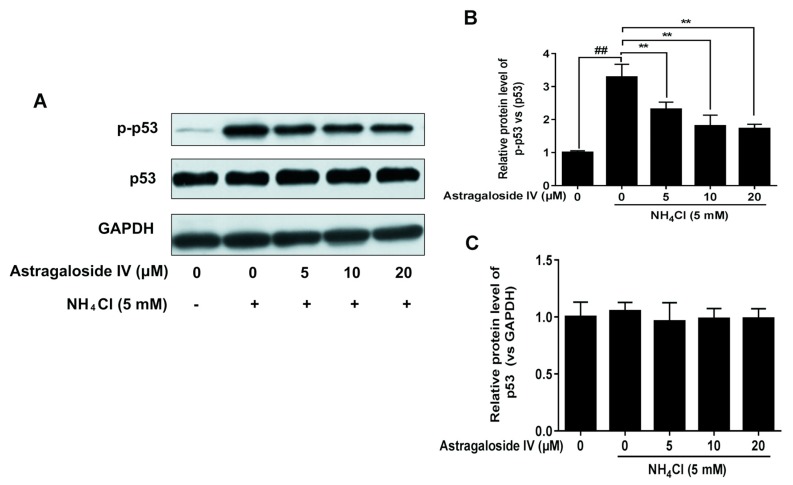
The protective effects of astragaloside IV on the protein levels of p-p53 and p53 induced by ammonia in MAC-T cells. The MAC-T cells were pretreated with different concentrations of astragaloside IV (0, 5, 10, and 20 μM) for 4 h, followed by NH_4_Cl (5 mM) treatment for 24 h. The relative protein levels of p-p53 and p53 were detected by Western blotting (**A**). Relative p-p53 (vs. p53) levels were analyzed by grey scanning (**B**). Relative p53 (vs. GAPDH) levels were analyzed by grey scanning (**C**). GAPDH was used as an internal reference for Western blotting analysis. The data are shown as mean ± SD. *n* = 3. **, *p* < 0.01. ## indicates a significant difference from untreated cells at *p* < 0.01.

The effects of astragaloside IV on p53 signaling pathways induced by ammonia in MAC-T cells were verified via Western blotting. As shown in Figure 3, ammonia significantly up-regulated the levels of p-p53, consistent with our previous research [3]. In the present study, astragaloside IV (at the concentrations of 5, 10 and 20 μM) significantly inhibited levels of p-p53 induced by ammonia. 

### 2.4. Effects of Astragaloside IV on mRNA Expression of Inflammatory Factors (IL-6 and IL-8) Induced by Ammonia in Bovine Mammary Epithelial Cells

**Figure 4:**
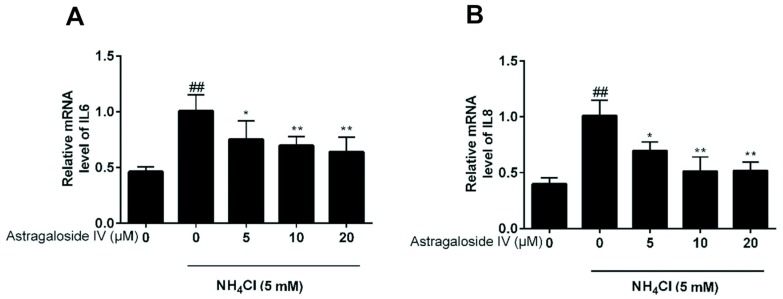
Effects of astragaloside IV on mRNA expression of inflammatory factors (*IL-6* and *IL-8*) induced by ammonia in MAC-T cells. The mRNA expressions of *IL-6* (**A**) and *IL-8* (**B**) were analyzed using quantitative real-time PCR. Data are represented as mean ± SD from three independent experiments. The data are shown as mean ± SD. *n* = 4. *, *p* < 0.05; **, *p* < 0.01. ## indicates a significant difference from untreated cells at *p* < 0.01.

The expressions of IL8 and IL-6 were detected using qPCR. The results showed that astragaloside IV at the concentrations of 5, 10, and 20 μM suppressed the production of IL8 and IL-6 in ammonia-treated MAC-T cells (Figure 4).

### 2.5. Effects of Astragaloside IV on mRNA Expression of Endoplasmic Reticulum Stress Markers (CHOP and GPR78) Induced by Ammonia in Bovine Mammary Epithelial Cells

**Figure 5:**
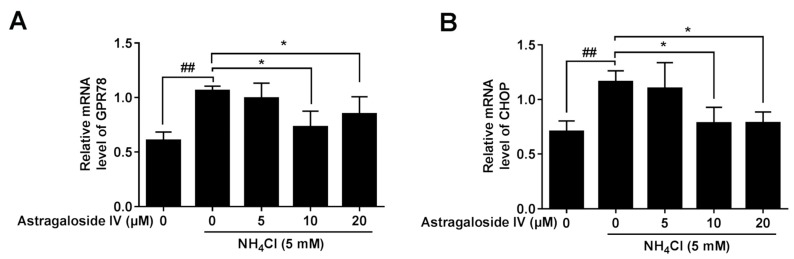
The protective effects of astragaloside IV on the mRNA expression of endoplasmic reticulum (ER) stress markers (CHOP and GPR78) induced by ammonia in MAC-T cells. The mRNA expressions of the endoplasmic reticulum stress markers *GRP78* (**A**) and *CHOP* (**B**) were analyzed using quantitative real-time PCR. Data are shown as mean ± SD. *n* = 4. *, *p* < 0.05; ## indicates a significant difference from untreated cells *p* < 0.01.

Ammonia significantly increased the mRNA expressions of the endoplasmic reticulum (ER) stress markers (CHOP and GPR78) in MAC-T cells (Figure 5). However, after pretreatment with astragaloside IV at the concentrations of 10 and 20 μM, the expressions of CHOP and GPR78 induced by ammonia were significantly suppressed.

### 2.6. The Cytoprotective Effects of Astragaloside IV against Oxidative Stress are Dependent on the Induction of Nrf2 in Bovine Mammary Epithelial Cells

**Figure 6:**
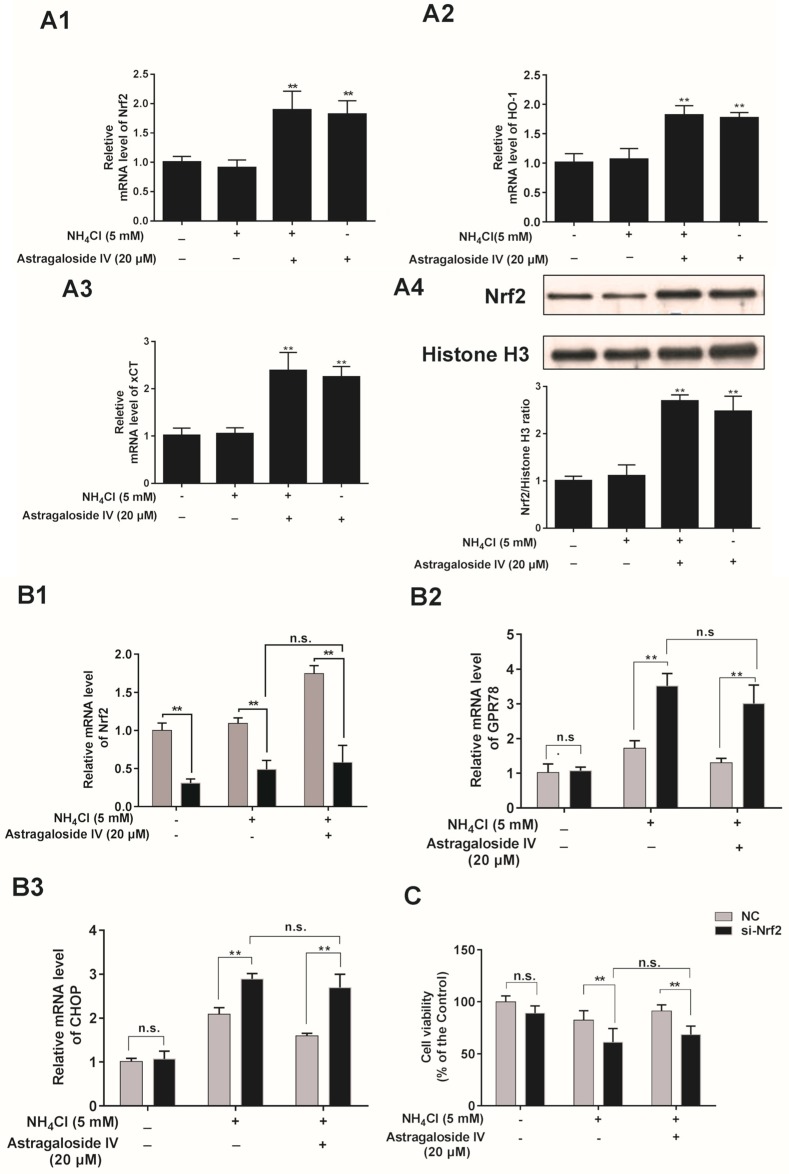
Astragaloside IV protects MAC-T cells against ammonia-induced oxidative stress by the activation of Nrf2. (**A**) Quantitative real-time PCR analysis of Nrf2 (**A1**), HO-1 (**A2**), and xCT (**A3**) mRNA in MAC-T cells pretreated with or without astragaloside IV for 4h and then treated with or without NH_4_Cl for 24 h. The nuclear protein level of Nrf2 (**A4**) was analyzed by Western blotting. Relative protein levels were analyzed by grey scanning. The data are shown as means ± SD, *n* = 3; **, *p* < 0.01. (**B**) The mRNA expressions of Nrf2(**B1**), GRP78(**B2**) and CHOP(**B3**) in MAC-T cells transfected with either an Nrf2 siRNA (Si-Nrf2-3) or a control siRNA (NC) for 16 h and pretreated with astragaloside IV for 4 h followed by NH_4_Cl treatment for an additional 24 h. (**C**) Cell viability assay in MAC-T cells transfected with either an Nrf2 siRNA or a control siRNA (NC) for 16 h and with or without astragaloside IV pretreatment for 4h followed by NH_4_Cl treatment for additional 24 h. The data are shown as mean ± SD. *n* = 6. **, *p* < 0.01. n.s. indicates no significance.

As shown in Figure 6A, astragaloside IV strongly upregulated mRNA expression of Nrf2, HO-1, and xCT under the ammonia-induced oxidative condition. The results of immunofluorescence staining showed that astragaloside IV stimulated Nrf2 protein levels in the cytoplasm and nucleus of MAC-T cells (Figure S3). In addition, the nuclear protein levels of Nrf2 were significantly increased with the astragaloside IV treatment (Figure 6A4). In addition, the role of Nrf2 in the cytoprotective effects of astragaloside IV on oxidative stress (ammonia induced) was detected by an Nrf2 siRNA transfection assay. The level of Nrf2 mRNA was significantly decreased after transfection into MAC-T cells with or without ammonia treatment compared to the cells transfected with a control siRNA. The level of Nrf2 mRNA was significantly decreased with or without pretreatment with astragaloside IV compared to the cells transfected with a control siRNA (Figure 6B). The expression of GPR78 and CHOP mRNAs in the Nrf2 knockdown group increased significantly compared with that in the cells transfected with a control siRNA and challenged with ammonia. In addition, an inhibitory effect of astragaloside IV on the mRNA expression of CHOP was not shown in the Nrf2 siRNA-transfected cells (Figure 6B). Furthermore, knockdown of Nrf2 abolished the protective effect of astragaloside IV against an ammonia-induced decrease in cell viability (Figure 6C).

### 2.7. Effects of Ammonia and Astragaloside IV Treatment on the AKT and ERK Signaling Pathways in Bovine Mammary Epithelial Cells

**Figure 7:**
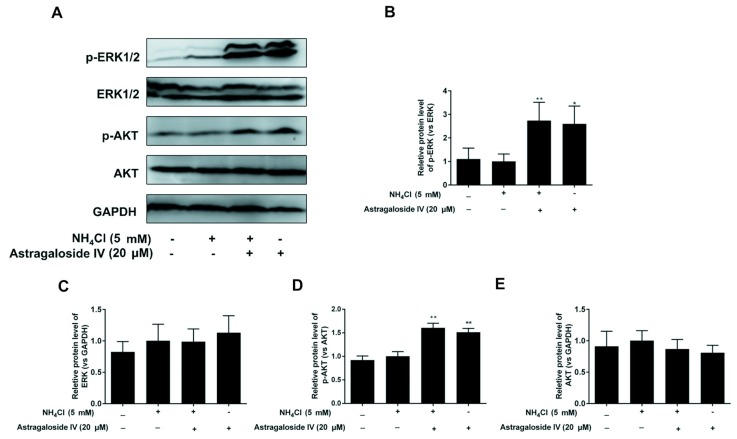
Astragaloside IV activates the ERK and AKT signaling pathways involved in survival of MAC-T cells against the ammonia-induced oxidative stress. Relative protein expression levels of p-ERK1/2, ERK1/2, p-AKT and AKT were detected by Western blotting analysis (**A**). Relative p-ERK (vs. ERK) levels were analyzed by grey scanning (**B**). Relative ERK (vs. GAPDH) levels were analyzed by grey scanning (**C**). Relative p-AKT (vs. AKT) levels were analyzed by grey scanning (**D**). Relative AKT (vs. GAPDH) levels were analyzed by grey scanning (**E**). The data are shown as means ± SD, *n* = 3; *, *p* < 0.05; **, *p* < 0.01.

To determine whether astragaloside IV is involved in activating the AKT and ERK signaling pathways in MACT cells (5 mM NH_4_Cl treatment), the levels of p-AKT and p-ERK were detected. As shown in Figure 7, the levels of p-AKT and p-ERK were increased by astragaloside IV pretreatment with or without ammonia exposure.

### 2.8. Activation of AKT and ERK Is Required for the Cytoprotective Effects of Astragaloside IV in Bovine Mammary Epithelial Cells

**Figure 8:**
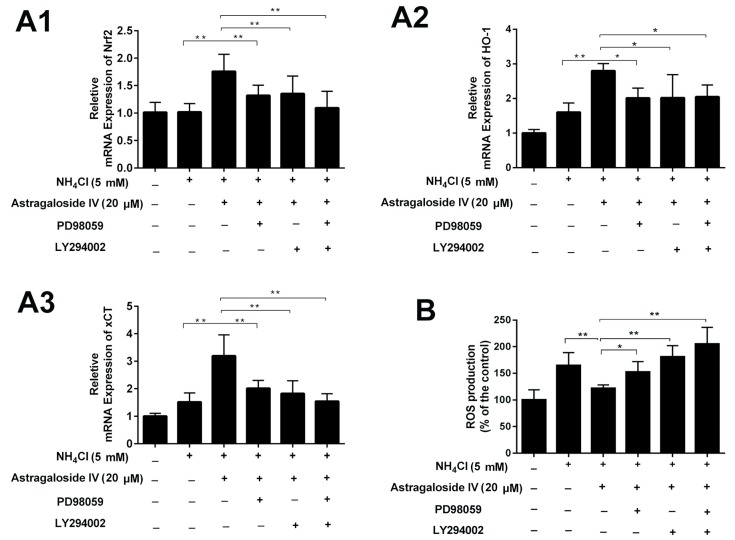
Activations of the ERK and AKT pathways mediate the cytoprotective effects of astragaloside IV against ammonia-induced oxidative stress in MAC-T cells. The cells were preincubated with or without specific kinase inhibitors LY294002 (3 μM) or PD98059 (10 μM) for 1 h and were then treated with or without astragaloside IV for another 4 h, followed by ammonia treatment for 24 h. The mRNA expressions of *Nrf2* (**A1**)*, HO-1* (**A2**) and *xCT* (**A3**) and the ROS level (**B**) were detected. The data are shown as means ± SD, *n* = 4; *, *p* < 0.05; **, *p* < 0.01.

Inhibitors LY294002 (for AKT signaling; 3 μM; Beyotime, Shanghai, China) and PD98059 (for ERK signaling; 10 μM; Beyotime, China) for various signaling pathways were used in MAC-T cell cultures to further study the signaling pathways for the cytoprotective function of astragaloside IV. Results showed that the astragaloside IV-induced an increase in the expression of Nrf2 and HO-1, and xCT was inhibited significantly by PD98059 and LY294002 (Figure 8A1–A3). In addition, PD98059 and LY294002 abrogated the ROS scavenging effects of astragaloside IV (Figure 8B).

## 3. Discussion

Ammonia at high concentrations has been implicated in oxidative stress [13,14]. In our previous study, we found that ammonia could induce oxidative stress and apoptosis in bovine mammary epithelial cells [3]. In the present study, we investigated the protective effects of astragaloside IV on bovine mammary epithelial cells exposed to ammonia. The beneficial effects of astragaloside IV may involve the regulation of Nrf2 by activating the ERK/MAPK and PI3K/AKT signaling pathways. Furthermore, astragaloside IV inhibits ammonia-induced cell apoptosis by inhibiting the p53 signaling pathways in MAC-T cells.

Dairy cows undergo rapid metabolic and physiological adaptations during calving and early lactation, hallmarked by reduced levels of blood ascorbates and increased lipids [15]. The mammary gland of dairy cows more easily suffers an oxidative state during this period [16]. The mammary epithelial cells of lactating cows accumulate a large amount of free radicals, like ROS [17]. Overproduction of ROS is widely recognized as an important index of oxidative stress [18]. In the present study, the level of ROS in the MAC-T cells increased significantly with ammonia treatment, suggesting that ammonia induces oxidative stress in bovine mammary epithelial cells, which further confirmed the results of our previous study [3]. Pretreatment of astragaloside IV decreased the level of ROS, suggesting a protective effect of astragaloside IV in the MAC-T cells against oxidative stress induced by ammonia.

The production of ROS and cell apoptosis are closely related [19]. The present study showed that the percentage of apoptotic cells increased significantly with ammonia treatment, a result which may be closely correlated with the overproduction of ROS. Again, this result is consistent with that of our previous study [3]. On the other hand, both the percentage of cell apoptosis and the level of ROS decreased under pretreatment with astragaloside IV compared with under treatment with ammonia alone, suggesting that astragaloside IV may suppress ammonia-induced apoptosis in MAC-T cells. Furthermore, the results showed that ammonia may induce mitochondrial damage and cell apoptosis in MAC-T cells as seen by the increased ratio of BAX/BCL2 in the ammonia-treated group. It has been shown that a high BAX/BCL2 ratio is associated with mitochondrial damage [20]. Interestingly, pretreatment with astragaloside IV decreased the levels of BAX, caspase 3, and phosphorylated p53 in MAC-T cells, indicating that astragaloside IV attenuated ammonia-induced apoptosis MAC-T cells by inhibiting the activation of the p53 signaling pathway. In addition, pretreatment with astragaloside IV decreased the ratio of BAX/BCL2 in MAC-T cells, indicating that astragaloside IV attenuated the ammonia-induced mitochondrial damage.

The endoplasmic reticulum plays an important role in cell survival and the synthesis of milk components in bovine mammary epithelial cells [21]. Studies have reported that oxidative stress likely induces damage and dysfunction of the endoplasmic reticulum [18,22,23]. In the present study, the expressions of mRNAs of the unfolded protein response regulators (CHOP and GRP78) were strongly upregulated by ammonia, indicating the ammonia-induced stress on endoplasmic reticula in MAC-T cells. However, astragaloside IV decreased the expressions of CHOP and GRP78, suggesting that astragaloside IV rescued the damage to the endoplasmatic reticulum induced by ammonia. 

Overproduction of ROS initiates inflammation [24]. In the present study, ammonia increased the levels of the main proinflammatory cytokines (IL-6 and IL-8) as well as the level of ROS, suggesting that ammonia-induced overproduction of ROS might play a key role in inflammatory responses. Astragaloside IV significantly inhibited the ammonia-induced increases in IL-6 and IL-8 levels in a dose-dependent manner, suggesting that astragaloside IV might protect against ammonia-induced inflammatory responses through its antioxidant potential.

To attain further insight into the antioxidative effect of astragaloside IV, expressions of genes encoding antioxidant/detoxificant enzymes (HO-1 and xCT) involved in cytoprotection against oxidative stress were observed in the present study. 

HO-1 is one of the major principal phase II enzymes promoting antioxidant activities [25]. In addition, xCT is a cystine transporter that regulates glutathione synthesis [25]. The expression of xCT and HO-1 is regulated by Nrf2-ARE [26]. Reports have shown that astragaloside IV protects primary cortical neuronal and mouse from oxidative stress through the Nrf2 pathway [27,28,29]. In the present study, astragaloside IV increased the expressions of Nrf2, HO-1, and xCT and promoted the nuclear protein level of Nrf2 in MAC-T cells, indicating that Nrf2 mediated the induction of HO-1 and xCT by astragaloside IV. Upon knockdown of Nrf2, the mRNA levels of CHOP and GPR78 were elevated, suggesting that the activation of Nrf2 reduced the rendoplasmic reticulum stress. 

Astragaloside IV activated the ERK and AKT signaling pathways involved in cell survival upon oxidative stress in neurons [30]. In the present study, it was also found that astragaloside IV could activate the ERK and AKT signaling pathways involved in MAC-T cell survival upon oxidative stress induced by ammonia. Furthermore, the astragaloside IV-induced gene expressions of Nrf2, HO-1, and xCT were inhibited by specific inhibitors of ERK and AKT, indicating that the ERK and AKT pathways play an important role in mediating the cytoprotective effects of astragaloside IV. 

**Figure 9:**
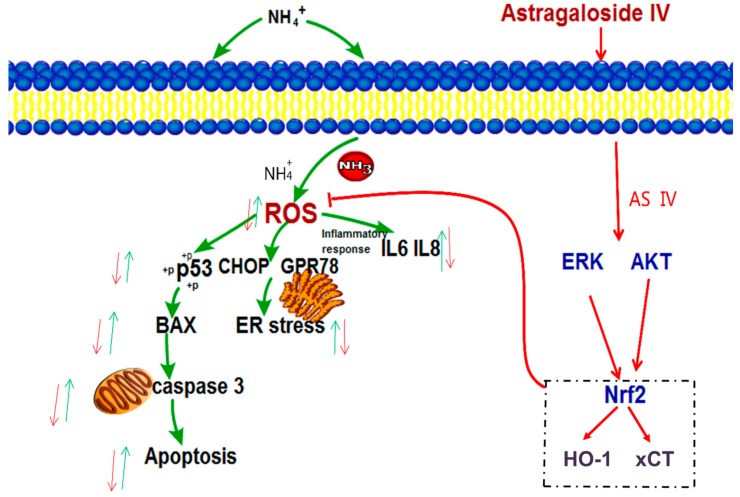
A putative mechanism of astragaloside IV alleviating ammonia-induced apoptosis and oxidative stress in bovine mammary epithelial cells. The green arrows indicate the effects of ammonia on MAC-T cells, and the red arrows indicate the effects of astragaloside IV on ammonia-induced apoptosis and oxidative stress in MAC-T cells.

In summary, this study provided strong evidence that astragaloside IV has potential cytoprotective effects against oxidative stress, apoptosis, and inflammatory responses in bovine mammary epithelial cells. Pretreatment with astragaloside IV protected MAC-T cells from ammonia-induced oxidative stress by suppressing ROS formation by activating the Nrf2-ARE pathway. Furthermore, astragaloside IV protected MAC-T cells against ammonia-induced cell apoptosis, inflammatory responses and endoplasmic reticulum damage. The protective effects of astragaloside IV against oxidative damage induced by ammonia in MAC-T cells might be mediated via activation of the ERK and AKT signaling pathways (Figure 9). The present work may provide a possibility for the therapeutic application of astragaloside IV in the protection of the mammary gland against ammonia exposure. This remains to be tested by an in vivo study of dairy cows.

## 4. Materials and Methods

### 4.1. Cell Culture and Treatment

The bovine mammary epithelial cell line (MAC-T) was maintained in DMEM/F12 1:1 (Hyclone, South Logan, UT, USA) medium supplemented with 10% fetal bovine serum (Gibco, Grand Island, NY, USA) at 37 °C with 5% CO_2_. NH_4_Cl (sigma, St. Louis, MO, USA) was dissolved in ddH_2_O. A new stock solution of 20 mM astragaloside IV (Yuanye biomart, Shanghai, China) was stored at room temperature. Astragaloside IV was dissolved in dimethyl sulfoxide (DMSO) (Sigma, St. Louis, MO, USA). The cells were pretreated for 4 h with astragaloside IV (at the concentrations of 0, 5, 10, and 20 µM), then cultured for 24 h (in medium supplemented with NH_4_Cl at the concentration of 5 mM) according to our previous study [3]. Cells were pretreated with astragaloside IV alone for 4 h, and NH_4_Cl was added to the same medium for 24 h.

### 4.2. Cell Viability Assay

The cell viability of the MAC-T cells was measured using a cell counting kit-8 (cck8 kit, Dojindo, Kumamoto, Japan). The cells were seeded in 96-well plates (10^4^ cells per well) for 6 h, then treated with astragaloside IV or NH_4_Cl at different levels. After 24 h of incubation, the optical density (OD) of cells was measured at 450 nm on a microplate reader (TECAN, Safire, Austria).

### 4.3. Detection of Intracellular ROS

The levels of intracellular ROS were detected using a reactive oxygen species assay kit (Beyotime, Shanghai, China). Briefly, after treatment, different treatments of MAC-T cells were washed with PBS (phosphate-buffered saline) and incubated with DCFH-DA at 37 °C for 30 min, followed by washing three times with PBS (pH = 7.2). The DCF fluorescence distribution of 20,000 cells was detected using a BD LSR flow cytometer (BD Biosciences, Franklin Lakes, NJ, USA) at an excitation wavelength of 488 nm and an emission wavelength of 535 nm.

### 4.4. Quantitative Measurement of Apoptosis by AnnexinV-FITC/PI Staining

The apoptotic rate of MAC-T cells was detected using FCM with annexin V-FITC and PI double labeling (Annexin V-FITC Apoptosis Detection Kit, Beyotime, Shanghai, China). Briefly, treated cells were harvested and incubated with AnnexinV-FITC and propidium iodide (PI) at room temperature for 10 min. Subsequently, samples were measured using a BD LSR flow cytometer (BD Biosciences, Franklin Lakes, NJ, USA).

### 4.5. RNA Isolation and Quantitative Real-Time PCR (qPCR)

Total RNA was isolated from cells using a TRIZOL Regent Kit (Takara, Tokyo, Japan) according to the manufacturer’s instructions. Approximately 1 µg of total RNA was used for cDNA syntheses using a standard reverse transcription kit (Takara, Tokyo, Japan). The inverse transcription process was as follows: 25 °C for 10 min, 42 °C for 15 min, and 85 °C for 5 min. The primers used (designed by AlleleID 6) are shown in Table 1 (synthesized in Comate Bioscience Co. Ltd., Changchun, China). qPCR was carried out in a 7500c real-time PCR detection system (Applied Biosystems, Carlsbad, CA, USA) with the SYBR premix EX Taq (TaKaRa). GAPDH was regarded as the control.

### 4.6. Transfection and Nrf2 Small RNA Interference

Three candidate siRNAs of Nrf2 and one negative control siRNA were synthesized by Ribobio (Guangzhou, China). The Nrf2 siRNA sequences were si-001: 5′-GGTATTTGACTTCAGTCAA-3′, si-002: 5′-CCAGAATTACAGTGTCTT-3′, and si-003: 5′-GGCTGAGACTAGTACAGTT-3′. The specificity and effectiveness of the three candidate siRNAs of Nrf2 were detected by quantitative analysis of the mRNA level of Nrf2 at 36 h after siRNA transfection. The results showed that the third candidate siRNA of Nrf2 was the most effective (80% knockdown, Figure S1). The MAC-T cells were transfected with the third siRNA of Nrf2 in subsequent experiments. The transfection experiments used the FUGENE^®^ HD (Roche, Basel, Switzerland) transfection reagent following the manufacturer’s instructions. Briefly, cells were cultured in 12 wells, and the transfection reagent was used to transfect Nrf2 siRNA at a concentration of 50 nM following the manufacturer’s instructions. The cells were treated with si-Nrf2 for 36 h in serum-free medium.

### 4.7. Western Blot Analysis

Total proteins from the MAC-T cells for different treatments were extracted using mammalian protein extraction reagent (M-PER, Thermo, Rockford, IL, USA). The nuclear proteins of cells were prepared by using the CelLytic NuCLEAR Extraction kit (Sigma-Aldrich, St. Louis, MO, USA). The protein concentration was detected using a BCA protein assay kit (Beyotime, Shanghai, China). Equal amounts of protein were loaded into each well, separated by 10% SDS-PAGE, and blocked with 5% bovine serum albumin dissolved in TBST for 1 h, and then incubated with primary antibodies (p53, p-p53, GAPDH, ERK, p-ERK, AKT and p-AKT; Bioworld, Shanghai, China; 1:1000) (Nrf2 and Histone H3, Abcam, Cambridge, MA, USA; 1:1000) at 4 °C for 12 h. The membranes were washed four times for 6 min each and then incubated with the appropriate second antibody conjugates (Abcam, Cambridge, MA, USA) or horseradish-peroxidase-conjugated protein antibody for 1 h at room temperature. Membranes were washed 4 times and then stained using DAB Horseradish Peroxidase (Color Development Kit Beyotime, Shanghai, China). The proteins were detected using the gel visualize Alpha Innotech and analyzed with the Tanon gel imaging system (Tanon, Shanghai, China). The influence of astragaloside IV on GAPDH expression was measured. There were no differences in the expression of GAPDH between the control group and AS IV group (Figure S2). GAPDH was regarded as the control (total protein) and Histone H3 was regarded as the control (nuclear protein).

### 4.8. Immunofluorescent Staining

The bovine mammary epithelial cells (MAC-T) were seeded in 24-well plates and grown until 50% confluence. Cells were then fixed with 10% formalin and blocked with Immunol Staining Blocking Buffer (Beyotime, Shanghai, China) followed by washing with TBST buffer (Triton X-100, 50 mM Tris, 0.15 mM NaCl, pH 7.6 containing 0.1% and Tween-20) three times. Cells were then incubated with a primary antibody for Nrf2 at a dilution of 1:200 (Abcam, Cambridge, MA, USA) at 4 °C for 12 h. After washing with TBST buffer, the cells were incubated with an FITC-labeled IgG diluted 1:1000 (Beyotime, Shanghai, China) in a special Secondary Antibody Dilution Buffer, (Beyotime, Shanghai, China). The nuclei of cells were stained with PI (Beyotime, Shanghai, China) for 15 min. The cells were viewed and analyzed using an Olympus fluorescence microscope (IX71, Tokyo, Japan).

### 4.9. Statistical Analysis

The data were analyzed with *t*-tests and one-way or two-way ANOVA, and the results are expressed as mean values ± standard deviation (SD). A value of *p* < 0.05 was considered to be statistically significant.

## Figures and Tables

**Table 1:** Primers for real-time quantitative PCR.

Primer Name	Primer Sequence (5′-3′)	Product Length (bp)
BAX	Sense Primer: TGCTTCAGGGTTTCATCC Anti-sense Primer: CTTCAGACACTCGCTCAG	116
BCL-2	Sense Primer: TTCTCCTGGCTGTCTCTG Anti-sense Primer: CTGCTTCTTGAATCTTCTGC	135
caspase 3	Sense Primer: GTTCATCCAGGCTCTTTGTG Anti-sense Primer: AAGGACTCATATTCTATTGCTACC	108
p53	Sense Primer: GAAGACCTACCCTGGCAATTAC Anti-sense Primer: AGAACAGCTTGTTAAGGGAAGG	104
GAPDH	Sense Primer: GTTCAACGGCACAGTCAAG Anti-sense Primer: TACTCAGCACCAGCATCAC	117
Nrf2	Sense Primer: CCAGCACAACACATACCATCAG Anti-sense Primer: CGTAGCCGAAGAAACCTCATTG	156
CHOP	Sense Primer: TGAACGACTCAAACAGGAAATC Anti-sense Primer: ACGCTAAGACCCTTTTCTATCG	248
GPR78	Sense Primer: GACCCTGACTCGGGCTAAAT Anti-sense Primer: TGGACAGCGGCACCATATG	243
HO-1	Sense Primer: GGCAGCAAGGTGCAAGA Anti-sense Primer: GAAGGAAGCCAGCCAAGAG	221
xCT	Sense Primer: GATACAAACGCCCAGATATGC Anti-sense Primer: ATGATGAAGCCAATCCCTGTA	136
IL6	Sense Primer: ATGCTTCCAATCTGGGTTC Anti-sense Primer: TGAGGATAATCTTTGCGTTC	269
IL8	Sense Primer: GCTGGCTGTTGCTCTCTTG Anti-sense Primer: GGGTGGAAAGGTGTGGAATG	126

## Supplementary Materials

The following are available online at http://www.mdpi.com/1422-0067/20/3/600/s1.

## Abbreviations

ROS	Reactive oxygen species
CHOP	C/EBP homologous protein
GRP78	Glucose-regulated protein 78
HO-1	Hemeoxygenase 1
Nrf2	Nuclear factor erythroid 2-related factor 2
