# Supplementary material for: Astragaloside IV Alleviates Ammonia-Induced Apoptosis and Oxidative Stress in Bovine Mammary Epithelial Cells
Source: Int J Mol Sci. 2019 Jan 30;20(3):600. doi: 10.3390/ijms20030600 (PMC6386910; doi:10.3390/ijms20030600)
Supplement: Supplementary file 1 [file ijms-20-00600-s001.pdf]

Supplementary materials

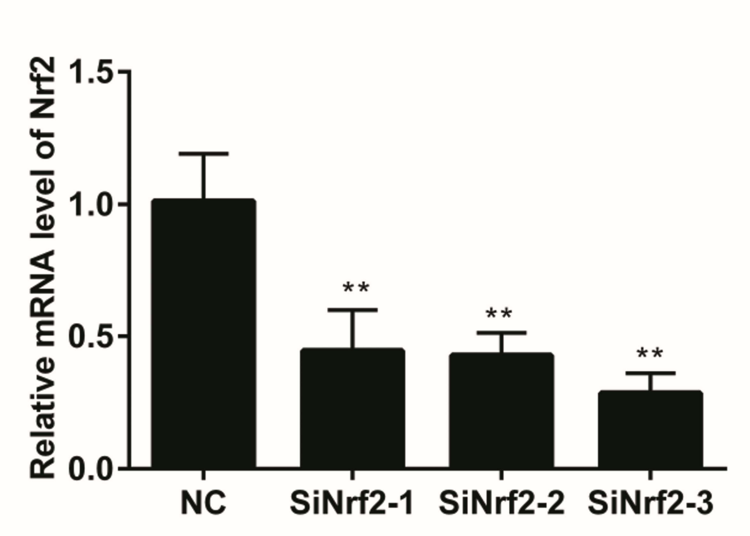

Figure S1. The Nrf2 mRNA expression of three different si-Nrf2 groups. The levels of Nrf2 mRNA of the three si-Nrf2 groups were significantly decreased compared to the cells transfected with a control siRNA (NC). The data are shown as mean ± SD. n = 4. *, P< 0.05; **, P< 0.01.

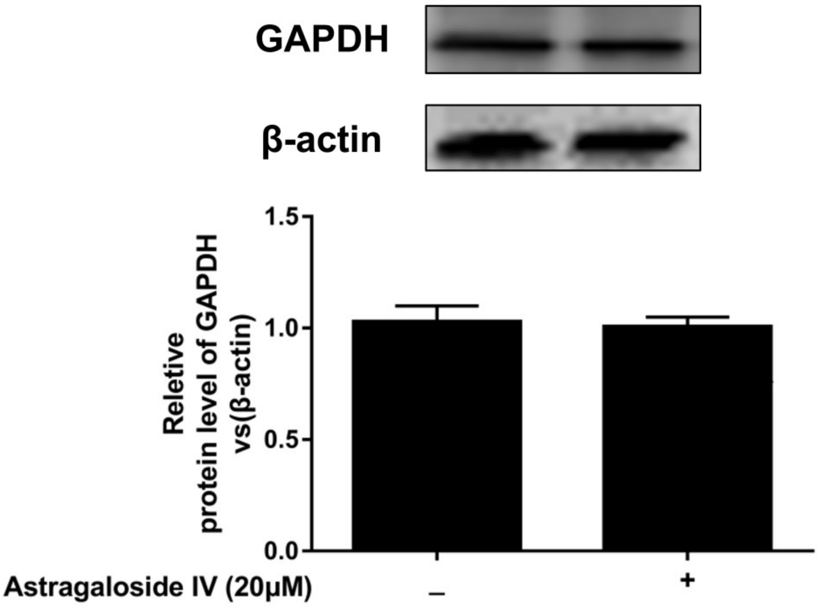

Figure S2. The effects of astragaloside IV on the protein levels of GAPDH. β-actin was used as an internal reference for Western blotting analysis. The proteins levels of GAPDH were detected by Western blotting. The relative GAPDH levels were analyzed by grey scanning. The data are shown as mean ± SD. n = 3.

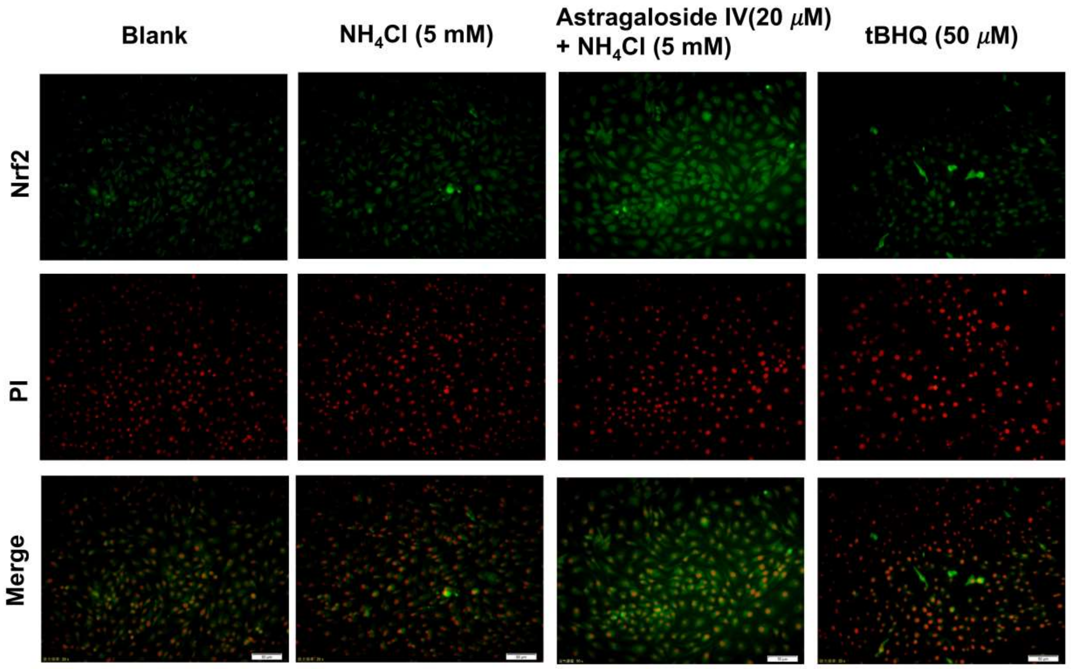

Figure S3. Immunofluorescence staining of Nrf2 in MAC-T cells treated with astragaloside IV (20 μM) for 24 h. PI staining was performed to stain the nucleus. Treatment of cells with tBHQ (40 μM) for 24 h served as a positive control for Nrf2 translocation.
